# Comparative transcriptome analysis of skin color-associated genes in leopard coral grouper (*Plectropomus leopardus*)

**DOI:** 10.1186/s12864-022-09091-6

**Published:** 2023-01-05

**Authors:** Hung-Yi Wu, Kao-Sung Chen, You-Syu Huang, Hern-Yi Hsieh, HsinYuan Tsai

**Affiliations:** 1 Department of Marine Biotechnology and Resources, National Sun Yat-Sen University, Kaohsiung City, Taiwan; 2 Planning and Information Division, Fisheries Research Institute, Council of Agriculture, Keelung, Taiwan; 3 Eastern Marine Biology Research Center, Taitung City, Taiwan; 4 Penghu Marine Biology Research Center, Penghu County, Magong, Taiwan; 5 Doctoral Degree Program in Marine Biotechnology, National Sun Yat-Sen University, Kaohsiung City, Taiwan

**Keywords:** *Plectropomus leopardus*, Skin color, Transcriptome, DEGs, qRT-PCR

## Abstract

**Background:**

The leopard coral grouper (*Plectropomus leopardus*) is an important economic species in East Asia-Pacific countries. To meet the market demand, leopard coral grouper is facing overfishing and their population is rapidly declining. With the improvement of the artificial propagation technique, the leopard coral grouper has been successfully cultured by Fisheries Research Institute in Taiwan. However, the skin color of farmed individuals is often lacking bright redness. As such, the market price of farmed individuals is lower than wild-type.

**Results:**

To understand the genetic mechanisms of skin coloration in leopard coral grouper, we compared leopard coral grouper with different skin colors through transcriptome analysis. Six cDNA libraries generated from wild-caught leopard coral grouper with different skin colors were characterized by using the Illumina platform. Reference-guided *de novo* transcriptome data of leopard coral grouper obtained 24,700 transcripts, and 1,089 differentially expressed genes (DEGs) were found between red and brown skin color individuals. The results showed that nine candidate DEGs (*epha2*, *sema6d*, *acsl4*, *slc7a5*, *hipk1*, *nol6*, *timp2*, *slc25a42*, and *kdf1*) significantly associated with skin color were detected by using comparative transcriptome analysis and quantitative real-time polymerase chain reaction (qRT-PCR).

**Conclusions:**

The findings may provide genetic information for further skin color research, and to boost the market price of farmed leopard coral grouper by selective breeding.

**Supplementary Information:**

The online version contains supplementary material available at 10.1186/s12864-022-09091-6.

## Background

Groupers are one of the most important economic fish species and popular aquaculture species in several Asian countries. About 47 species of grouper are cultivated in East and Southeast Asia [1], such as *Epinephelus lanceolatus*, *E. coioides*, *E. malabaricus*, and *Plectropomus leopardus*. The leopard coral grouper (*P. leopardus*) is belongs to the *Plectropomus* genus of the Serranidae family, which is mainly distributed in the western Pacific Ocean from the south of Japan to Australia, and the east of the Caroline Islands [2]. In nature, leopard coral grouper with different skin colors including red and brown have been found around the sea of Taiwan, Penghu, Okinawa, and the South China Sea [3–5]. In recent years, studies showed that overfishing and the destruction of spawning aggregations significantly declined leopard coral grouper population in the Philippines and Australia [6–8]. Even though the Red Book of the International Union for Conservation of Nature (IUCN) currently lists leopard coral grouper as the least concern [9], it is still essential to establish fishery management and conservation strategies to prevent leopard coral grouper from becoming a species of high-risk level in the future.

Coloration plays an important role in many creatures, which is associated with thermoregulation, social communication, predator avoidance, camouflage, protection from radiation, and selective mating [10, 11]. Previous studies indicated that coloration of teleost fish is determined by six types of pigment cells, including melanophores (black and dark brown), xanthophores (yellow), erythrophores (red and orange), iridophores (reflective), leucophores (white), and cyanophores (blue) [12–14]. Studies also suggested that cAMP (cyclic adenosine monophosphate), MAPK (mitogen-activated protein kinase), PI3k/Akt (phosphatidylinositol 3-kinase-Akt), and Wnt (wingless-type MMTV integration site) signaling pathways are involved in melanin-synthesis related pathways in fish [15–18]. The leopard coral grouper has bright red skin color, which is different from the other species in the same genus. Due to bright red being associated with good luck and happiness in Chinese culture [19], the net price of bright red leopard coral groupers is approximately US$7 per kilogram higher than the brown individuals in the Asian market [5]. As such, for leopard coral grouper, skin color is an important economic trait. Although artificial breeding has been successfully established for leopard coral grouper by Fisheries Research Institute in Taiwan, mostly the skin color of farmed individuals is gray and brown. Several factors, such as habitats, physiological response, food, and genetics, were associated with skin coloration [20–22]. Feed additives and breeding approach have been proposed in order to improve their skin color performance [23, 24]. Previous studies showed that the feed additives of carotenoids contain astaxanthin [4, 25, 26], enhancing the skin color of fish appearing red pigmentation. Besides, gene regulation associated with skin color is also a critical factor. Candidate carotenoid-related genes, including *BCO2*, *LRP11*, *ANGPTLs*, *ALAS*, *PDIL*, *MED12*, *SOX*, *FAX*, *FATP*, *SCD*, and *LDLRA*, were involved in carotenoid metabolism and significantly linked to the skin coloration of leopard coral grouper [5, 22, 27, 28]. Several miRNAs including miR-215, miR-2188, miR-194, miR-122, and novel-m0118 may also play a potential role in regulating skin color in the red-colored leopard coral groupers [29].

In 2020, the whole genome sequence of leopard coral grouper has been published [5, 30, 31], and results showed that the length of whole genome sequence is smaller than other grouper species, implying the evolutionary ancient status of leopard coral grouper genome compared to other grouper species [5]. Genetic markers, such as microsatellite loci, were frequently applied to understand the genetic diversity of wild-type leopard coral grouper, which is also useful for breeding program development and fishery management [32–35]. By calculating the heterozygosity ratio of the microsatellite locus of farmed fish, Yang et al. (2020) indicated that the heterozygosity ratio of the leopard coral grouper is 0.42% [5], which is higher than the red-spotted grouper (*E. akaara*) (0.38%) [36], Murray Cod (*Maccullochella peelii*) (0.11%) [37], pike-perch (*Sander lucioperca*) (0.14%) [38], *Triplophysa tibetana* (0.1%) [39], male grass carp (*Ctenopharyngodon idellus*) (0.25%) and female grass carp (0.09%) [22], indicating that leopard coral grouper is still a low-level anthropogenic selection species among aquaculture species, and with a high degree of ethnic genetic divergence.

**Fig. 1:**
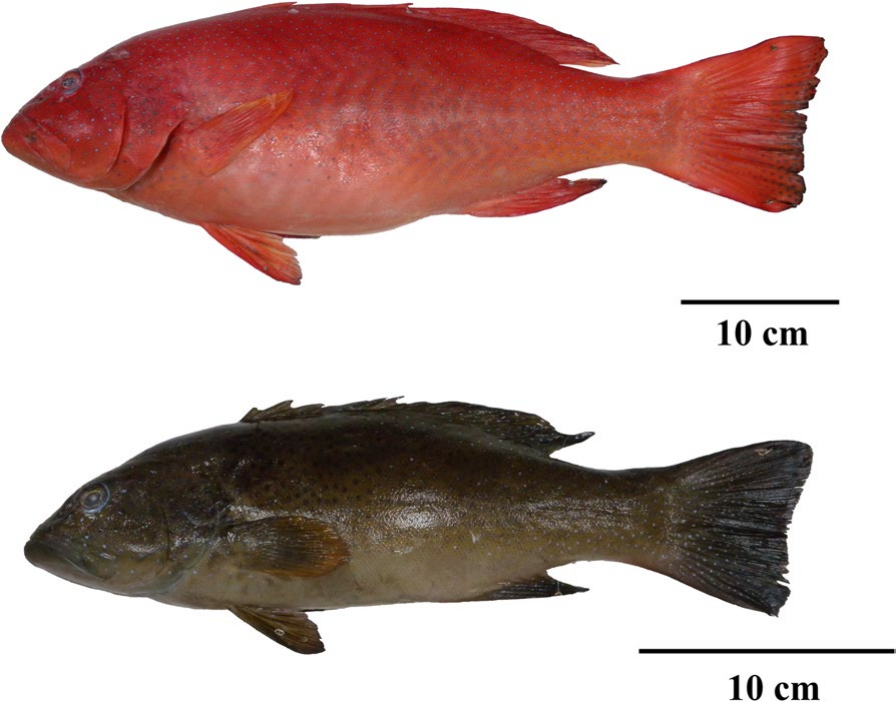
Leopard coral grouper (*Plectropumus leopardus*). Different skin colors, including red and brown individuals, were involved in this study. The fish were collected from Penghu Sea in Taiwan. Scale bars: 10 cm

Transcriptome analysis has been widely used to reveal gene expression affecting the skin coloration in non-model organisms, such as common carp (*Cyprinus carpio*) [17, 40, 41], crucian carp (*Carassius auratus*) [42], crimson snapper (*Lutjanus erythropterus*) [43], and catfish-like loach (*Triplophysa siluroides*) [44]. However, the molecular mechanism of skin coloration is still poorly understood in the Serranidae family. In this study, we characterized the transcriptome sequences of leopard coral grouper with different skin colors (red and brown) collected from Penghu Sea in Taiwan (Fig. 1). We aimed to (i) conduct transcriptome analysis of leopard coral groupers with different skin colors; (ii) validate differentially expressed genes (DEGs) associated with different skin colors of leopard coral groupers from other populations. The purpose of this study was to reveal novel information on candidate DEGs related to skin coloration between red and brown leopard coral groupers. Ultimately, the findings could provide useful genetic information for the artificial breeding of leopard coral grouper with different skin colors.

## Results

### Illumina sequencing and genome-guided de novo transcriptome assembly

The transcriptome of six leopard coral groupers, including three red individuals and three brown individuals, were sequenced, respectively (Table 1). The general statistics of the transcriptome sequencing results were presented in Table 1. A total of 47.5 Gb Illumina raw reads were obtained. After the quality control step, 42.6 Gb cleans reads were yielded. The total number of clean reads for six samples were 36,423,012, 40,269,914, 67,288,760, 74,867,838, 39,202,166, and 40,900,758, respectively. The total number of bases for six samples were 5,243,362,538, 5,884,011,219, 9,473,785,507, 10,512,398,825, 5,643,111,214, and 5,888,656,708, respectively. Clean reads with Q20 and Q30 were more than 92%, and the average GC content was 47.5%. The results showed that high-quality reads were used for transcriptome assembly in this study (Table 1).

**Table 1:** Summary statistics of sequencing data

Sample	skin color	Raw reads	Clean reads	Bases (bp)	Q20 (%)	Q30 (%)	GC (%)
R1	Red	39,045,732	36,423,012	5,243,362,538	97.12	92.33	47.62
R2	Red	43,187,736	40,269,914	5,884,011,219	96.96	92.00	46.94
R3	Red	68,533,404	67,288,760	9,473,785,507	98.63	95.06	48.36
B1	Brown	76,223,858	74,867,838	10,512,398,825	98.58	94.88	46.97
B2	Brown	42,298,544	39,202,166	5,643,111,214	97.08	92.22	48.11
B3	Brown	43,232,198	40,900,758	5,888,656,708	97.25	92.56	47.17

**Fig. 2:**
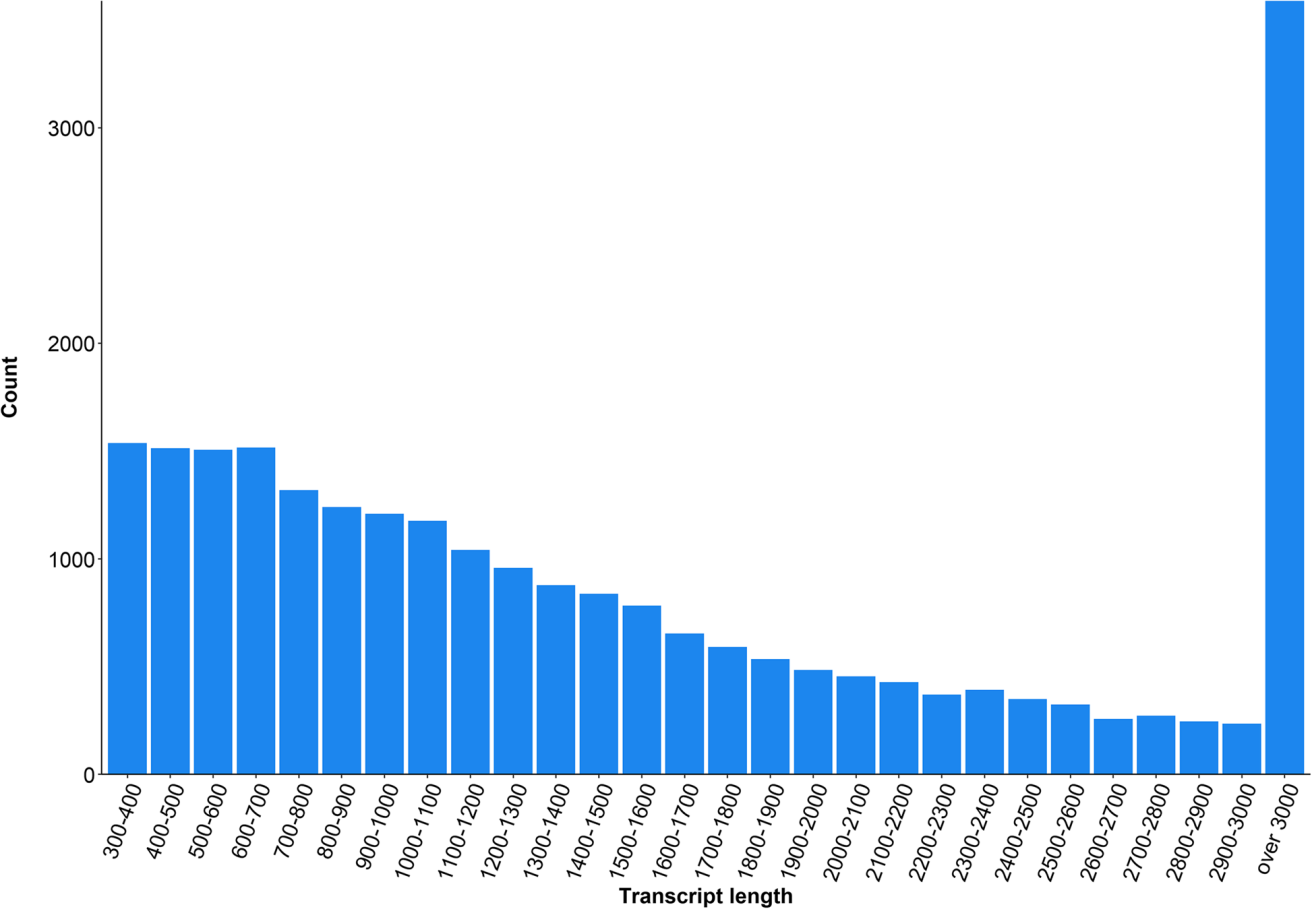
Length distribution of all transcripts

The final contig assembly produced 24,700 transcripts with an N50 length of 2,496 bp, an average contig of 1,776 bp, a medium length of 1,230 bp, and a GC content of 46.7% using CD-HIT-EST. The length distribution of all the transcripts was shown in Fig. 2. The longest and shortest contigs were 65,925 bp and 306 bp, respectively. There were 14,860 transcripts longer than 1,000 bp, accounting for 60% of complete data (Fig. 2).

### Functional annotation

**Fig. 3:**
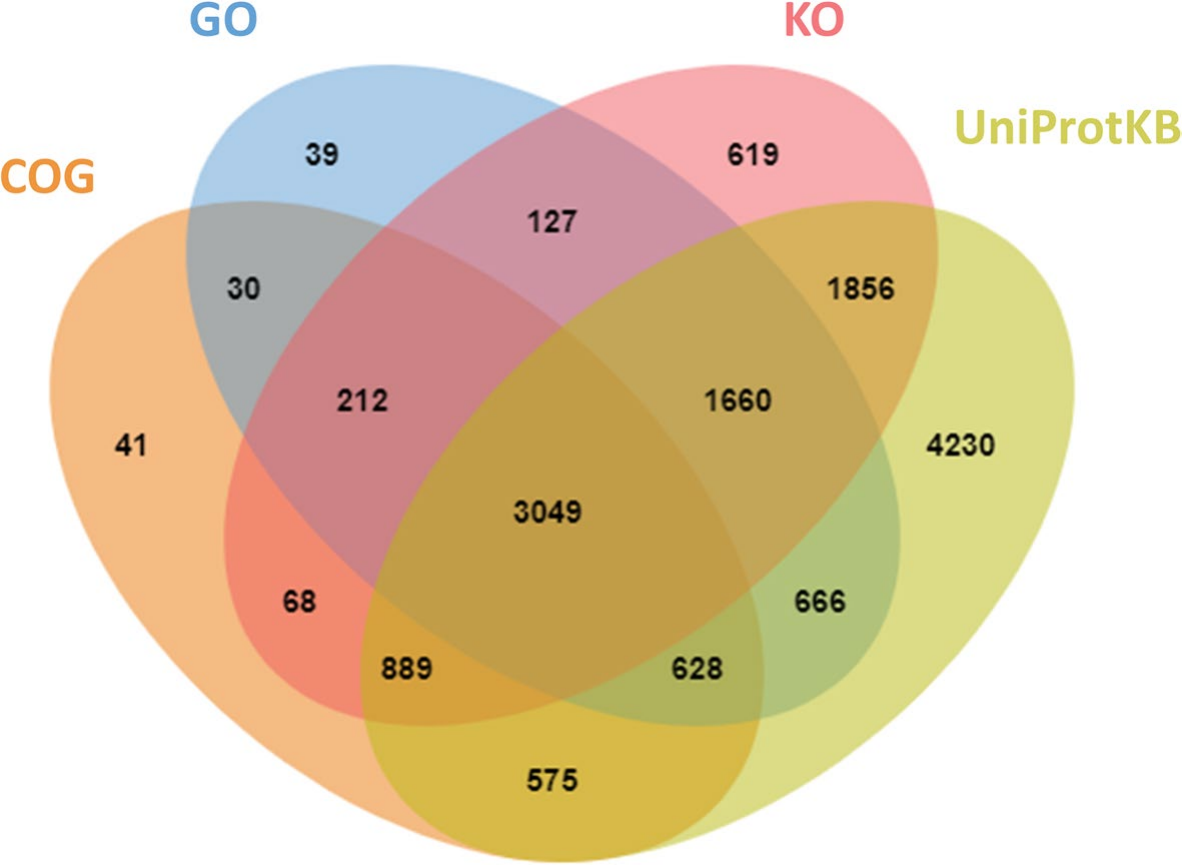
Venn diagram of annotation results based on UniProtKB, GO, COG, and KO databases, respectively. Venn diagram showed common and specific genes from the UniProtKB, GO, COG, and KO databases

To annotate these assembled transcripts, which were based on UniProt Knowledgebase (UniProtKB), Cluster of Orthologous Groups database (COG), Gene Ontology (GO), and Kyoto Encyclopedia of Genes and Genome Orthology (KO), including 14,171 genes, 5,492 genes, 6,411 genes, 8,480 genes accounting for a total of 24,700 genes, were obtained, respectively. 3,049 common genes were annotated by UniProtKB, COG, GO, and KO (Fig. 3). In the COG, the predominant categories were general function prediction only (20%), followed by cell cycle control, cell division, chromosome partitioning (17%), and function unknown (12%) (Additional file 1: Fig. S1). GO was divided into three major function categories: molecular functions (52%), cellular components (8%), and biological processes (40%) (Additional file 1: Fig. S2). KO classification obtained seven categories including 47 pathways. Lipid metabolism (108 genes) and transport and catabolism (128 genes) were the most abundant term in the categories of metabolism and cellular processes (Additional file 1: Fig. S3).

### Genetic relationship between different skin color fishes

**Fig. 4:**
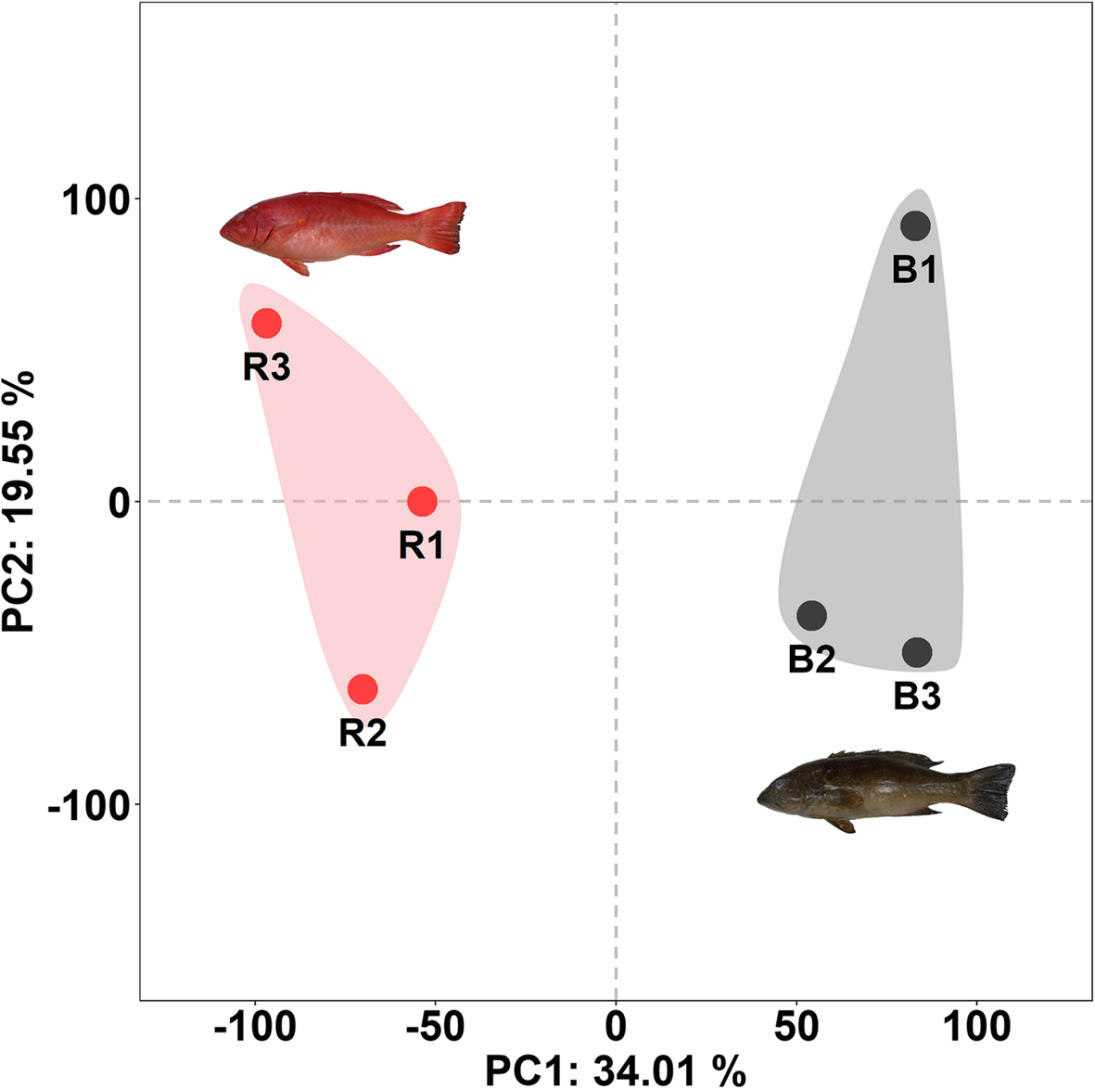
Principal component analysis of TMM expression in fish with different skin colors. Red skin fish: R1, R2, and R3. Brown skin fish: B1, B2, and B3

**Fig. 5:**
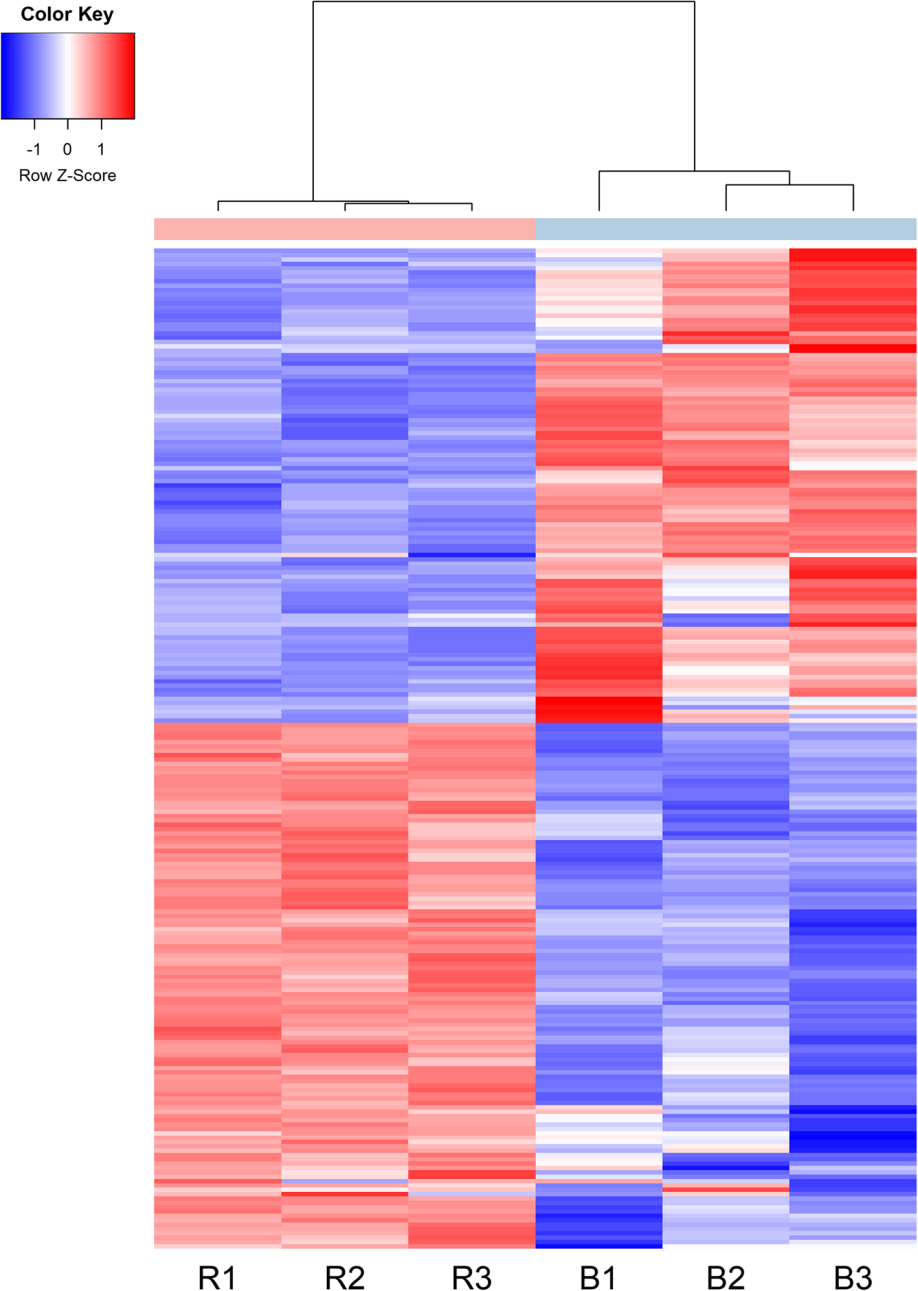
Heatmap analysis of DEGs. Transcriptome analysis showed top 230 DEGs (115 up regulation and 115 down regulation) related to skin color in red (R1, R2, and R3) and brown (B1, B2, and B3) individuals, respectively. The completed name of DEGs were given in Additional file 2: Table S1

The principal component analysis (PCA) plot showed that the red (R1, R2, and R3) and brown individuals (B1, B2, and B3) were clearly separated with main principal component (PC) scores as follows: PC1 = 34.01% and PC2 = 19.55% (Fig. 4). Heatmaps based on the trimmed mean of M-values (TMM) expression of top 230 DEGs (115 up regulation and 115 down regulation) were generated from six transcriptome sequencing samples. Results showed that the transcripts count of red individuals (R1, R2, and R3) was different from that of the brown individuals (B1, B2, and B3) in Fig. 5 and Additional file 2: Table S1.

### Differential expression genes

To analyze the DEGs between the red group (R1, R2, and R3) and the brown group (B1, B2, and B3). There were 1,089 DEGs (613 up-regulated and 476 down-regulated DEGs) obtained based on an absolute value of log_2_ fold change (FC) ≥ 2 and false discovery rate (FDR) < 0.05 (Additional file 1: Fig. S4). For the GO analysis, up-regulated and down-regulated DEGs were highly enriched in GO terms of protein binding, plasma membrane, nucleus, cytoplasm, and cytosol (Additional file 1: Fig. S5). For the KEGG pathways enrichment analysis, there were significantly enriched signal pathways, including Jak-STAT, cAMP, Rap1, TGF-beta, and PI3K-Akt signaling pathway in up-regulated DEGs. The most significantly enriched KEGG pathways were hypertrophic cardiomyopathy (HCM), dilated cardiomyopathy (DCM), and cardiac muscle contraction in down-regulated DEGs (Additional file 1: Fig. S6).

### Validation of differentially expressed genes by qRT-PCR

**Fig. 6:**
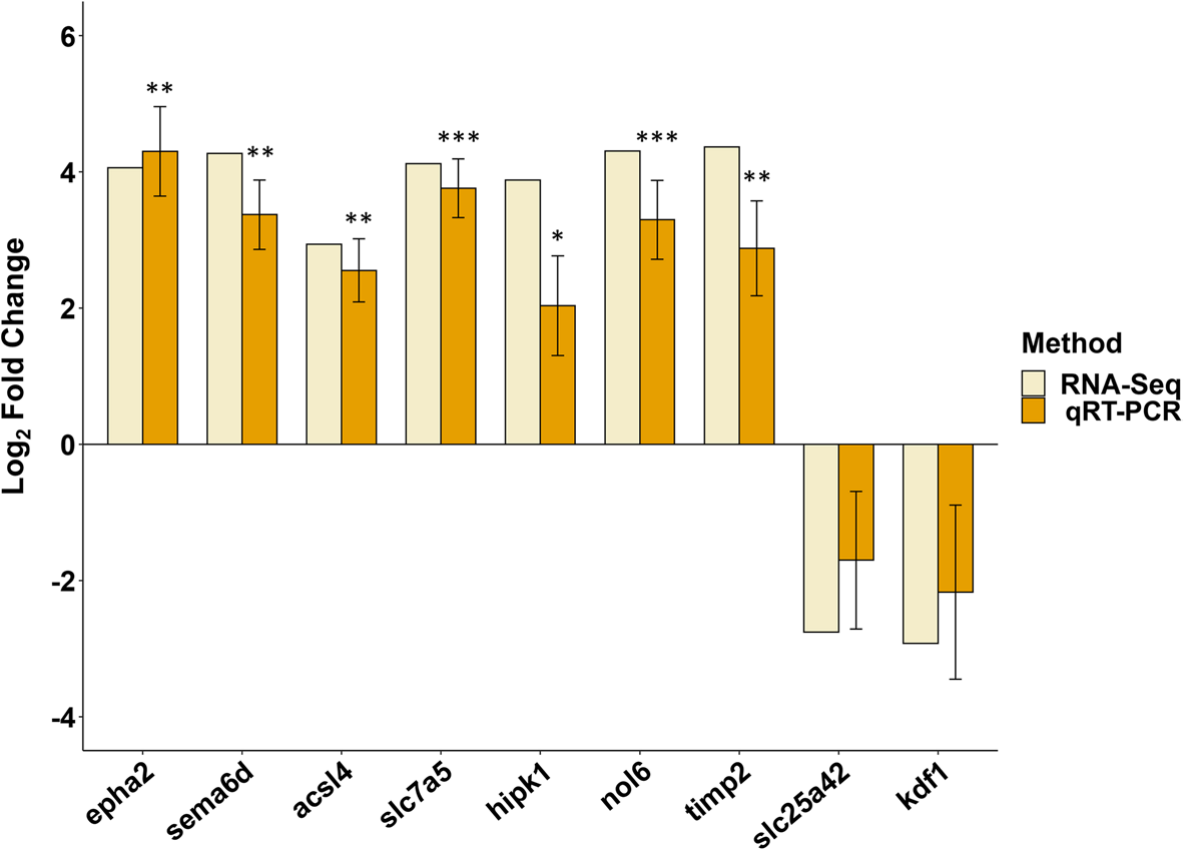
Comparison of gene expression patterns obtained using comparative transcriptome analysis and qRT-PCR. Nine genes were identified as DEGs between brown (control group) and red individuals (experimental group) (*n* = 4 for each group). Expression of target genes was normalized to β-actin as a reference gene. The Y-axis shows the relative mRNA expression levels (means ± SD). Statistically significant differences compared to control group are presented, with *: *P* < 0.05; **: *P* < 0.005; ***: *P* < 0.001

**Fig. 7:**
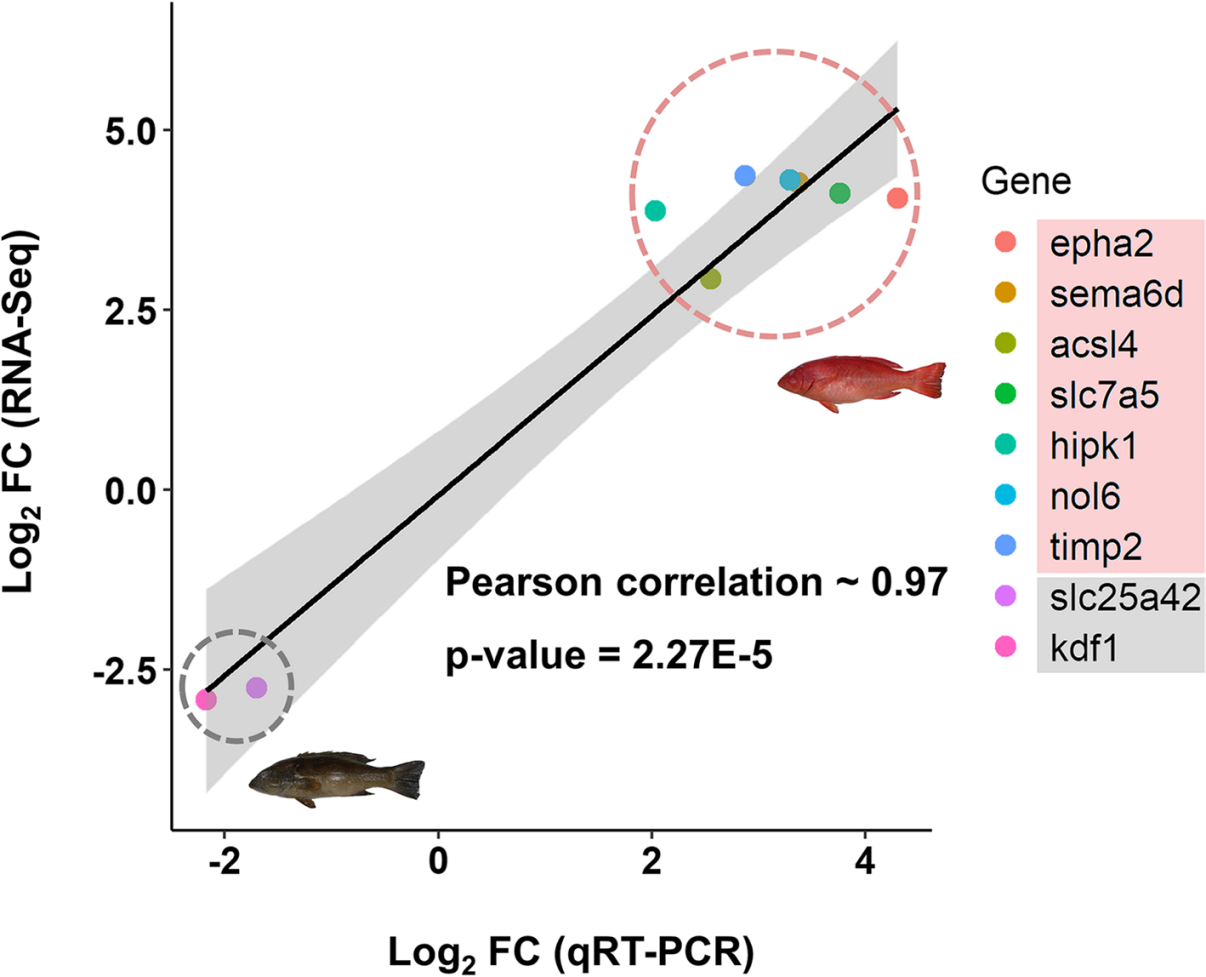
Correlation of gene expression analysis between comparative transcriptome analysis and qRT-PCR. Gene expression of *epha2*, *sema6d*, *acsl4*, *slc7a5*, *hipk1*, *nol6*, and *timp2* increased in red individuals, and gene expression of *slc25a42* and *kdf1* decreased in red individuals. qRT-PCR and RNA-Seq fold change values were highly correlated (Pearson correlation ~ 0.97 and *p*-value = 2.27E-5)

To investigate the DEGs associated with the red and brown skin coloration of leopard coral grouper, nine DEGs (FDR < 0.05 and |log_2_FC| ≥ 2) may associate with the skin coloration were chosen for qRT-PCR analysis (Table 2). These nine DEGs included eph receptor A2 (*epha2*), semaphorin 6D (*sema6d*), acyl-CoA synthetase long-chain family member 4 (*acsl4*), solute carrier family 7 member 5 (*slc7a5*), homeodomain interacting protein kinase 1 (*hipk1*), nucleolar protein 6 (*nol6*), TIMP metallopeptidase inhibitor 2 (*timp2*), solute carrier family 25 member 42 (*slc25a42*), and keratinocyte differentiation factor 1 (*kdf1*). The primers for qRT-PCR analysis were given in Table 3. The gene expression of *epha2*, *sema6d*, *acsl4*, *slc7a5*, *hipk1*, *nol6*, and *timp2* were increased, and the gene expression of *slc25a42* and *kdf1* were decreased. The log_2_ FC value of *epha2* in red individuals was significantly higher than brown individuals by 17 folds (Fig. 6). There was a high correlation between RNA-Seq and qRT-PCR with Pearson’s correlation of 0.97 (Fig. 7).

**Table 2:** Candidate genes involved in skin color between red and brown individuals

Gene_ID	FPKM (red)	FPKM (brown)	FDR	Fold change (log_2_)	Annotation	Gene symbol
*Up-regulated candidate DEGs associated with skin color in red fish*						
maker_0147_ augustus-0.133	337.99	30.72	1.15E-15	4.06	eph receptor A2	*epha2*
maker_0315_ augustus-0.115	166.45	15.29	4.60E-10	4.12	solute carrier family 7 member 5	*slc7a5*
maker_0681_ snap-0.96	296.75	60.74	3.20E-07	2.94	acyl-CoA synthetase long chain family member 4	*acsl4*
maker_0101_ snap-0.156	2608.51	196.59	9.38E-12	4.31	nucleolar protein 6	*nol6*
augustus_0141_ processed-1.34	303.58	31.55	9.05E-12	3.88	homeodomain interacting protein kinase 1	*hipk1*
maker_0248_ augustus-0.220	3725.68	281.98	5.67E-10	4.37	TIMP metallopeptidase inhibitor 2	*timp2*
maker_0156_ augustus-2.210	521.19	41.71	5.80E-13	4.27	semaphorin 6D	*sema6d*
*Up-regulated candidate DEGs associated with skin color in brown fish*						
maker_0129_ snap-3.241	11.56	118.54	1.85E-08	-2.76	solute carrier family 25 member 42	*slc25a42*
augustus_0124_ processed-0.83	4.94	55.99	6.88E-07	-2.92	keratinocyte differentiation factor 1	*kdf1*

**Table 3:** List of primers used for qRT-PCR.

Gene	Primer name	Sequence (5’-3’)	Amplicon size (bp)
*β-actin*	β-actin-F β-actin-R	TACGAGCTGCCTGACGGACA GGCTGTGATCTCCTTCTGCA	240 bp
*hipk1*	hipk1-F hipk1-R	GGCCAGATTGAGGTGGGTATC AATACCAGGCATGTGTGACCTT	113 bp
*epha2*	epha2-F epha2-R	GATGGACACGCGTGGATCAAA CTCCAAACGACCTCGCTTCA	106 bp
*slc7a5*	slc7a5-F slc7a5-R	AAGCCGATCGCACACTCCTT AGCGACCGATCAAGGTGAATA	109 bp
*acsl4*	acsl4-F acsl4-R	AAAAAGTGCACACAGAAGGCTAC CAATGGGCCGTTCCATATTCTC	102 bp
*nol6*	nol6-F nol6-R	GCCATTGACTAGGGTGAGATGT CGGCATGTACTATGATGCCCT	118 bp
*timp2*	timp2-F timp2-R	GTGCAGCGGATGATCTTGCAATC ATCACACTGTGTCACCTCATTCA	110 bp
*sema6d*	sema6d-F sema6d-R	GTCTGGTACTCCTTTACAGGGC CGAGGCATGTCCACAAGGAT	109 bp
*slc25a42*	slc25a42-F slc25a42-R	ATAAGGGTCATGCCCTACGC GAGGCAGAGTTTTTCCCTGGTA	100 bp
*kdf1*	kdf1-F kdf1-R	TCATGAGCAGTGACGTTCCA CTCATCTTCCTCGCCAGTTCA	73 bp

## Discussion

Leopard coral grouper is a valuable marine species in coral reef ecosystems, and an excellent research model for exploring its special skin color variations [5]. Fish skin coloration is a complex trait that is determined by genetic, cellular, physiological, and environmental factors [16]. The skin color in the fish, such as red leopard coral grouper and red tilapia, is the major factor of commercial value determination in Asian countries [45, 46]. Therefore, it is essential to understand the genetic mechanism of color variations in these aquaculture species. To date, several investigations have reported many candidate genes associated with color changes through RNA-Seq in different non-model teleost fishes, such as common carp, red tilapia, and Taiwanese loach [41, 42, 47, 48], as well as in model organisms such as zebrafish (*Danio rerio*) and medaka (*Oryzias latipes*) [49, 50]. Fang et al. (2021) and Zhang et al. (2017) indicated that the candidate skin color genes were related to tyrosine and pteridine metabolism, melanogenesis, ion change, apoptosis, and autophagy [42, 45]. Previous studies reported erythrophore/xanthophore synthesis (*slc7a11* and *slc24a5*), *try*, *tryp1*, *dct*, *mitfa*, and *sox10* are well-recognized melanophore-markers in red crucian carp and red tilapia [42, 51]. Many *SLC* genes regulating the transport of substances on the cell membrane have been involved in skin pigmentation in humans and fish, such as *slc45a2, slc24a5*, and *slc7a11*. *slc7a11* encodes the cystine/glutamate exchanger xCT that increases pheomelanin synthesis in red skin formation [42, 51, 52]. *slc24a5* plays an essential role in regulating melanophore development in several vertebrates, such as zebrafish and common carp, so knockdown of the *slc24a5* could block melanin synthesis to generate albino or golden phenotype [53, 54]. *slc45a2* participates in the membrane-associated transporter protein (MATP), which is likely involved in intracellular processing and trafficking of melanosomal proteins [55].

In the present study, we performed a comparative transcriptomic analysis between the red and brown skin of leopard coral grouper, and 1,089 significant DEGs were identified. Through comparative transcriptome analysis and qRT-PCR, we found that mRNA expression levels of *epha2*, *sema6d*, *acsl4*, *slc7a5*, *hipk1*, *nol6*, and *timp2* were greater in the red individuals compared to brown individuals, and that mRNA expression levels of *slc25a42* and *kdf1* were lower in the red individuals compared to brown individuals. Two candidate *SLC* genes (*slc7a5* and *slc25a42*) were identified as significant DEGs for skin color formation in leopard coral groupers. *slc7a5* was an up-regulated DEG in red individuals, and *slc25a42* was an up-regulated DEG in brown individuals. *slc7a5* is an amino acid transporter that regulates the mTOR pathway, enhancing pheomelanin synthesis through activating xCT expression in mice [56]. The SLC25 family member is the largest solute transporter family in humans and transports solutes across the inner membrane of mitochondria [57]. *slc25a38* contributed to red color development in spider mites [58], which may imply that *slc25a42* was the potential pigmentation-related gene in leopard coral grouper.

*epha2* is a tyrosine kinase, which belongs to the family of Eph receptors. It was reported that *epha2* inhibits MAPK and AKT pathways in human lens epithelial (HLE) cells [59], so the gene may prevent the MAPK pathway from activating the downstream gene of melanocyte inducing transcription factor (MITF), which is well known to participate in the enzymatic conversion of tyrosine to melanin [42, 59]. *acsl4* encodes a protein associated with lipid biosynthesis and fatty acid degradation [60]. Recent studies have indicated that *acsl4* plays a critical role in activating ferroptosis that leads to cell death by iron-dependent lipid peroxidation, and also inhibiting melanin synthesis [61]. *epha2* and *acsl4* were up-regulated DEGs, which may inhibit melanin synthesis in red skin color individuals. In addition, melanin plays an essential role to protect animals from UV radiation and environmental challenges. Keratinocyte-derived factors are involved in regulating the proliferation and melanogenesis in mammals [62, 63]. In our results, *kdf1*, a kind of keratinocyte-derived factor, had a higher expression in brown skin color individuals.

Taken together, most skin color-associated genes found in the study are involved in melanin synthesis which has been reported in previous studies. The genetic mechanisms of pigment cells are similar between fish and humans, and some skin color-associated genes in fish models, such as *slc25a42* and *MATP*, have contributed to understanding the genetic mechanism in human skin [64].

## Conclusions

This study conducted comparative transcriptome analysis in different skin colors of leopard coral groupers, and results showed that red individuals were different from brown individuals. The results of qRT-PCR showed that nine candidate genes were associated with the formation of skin coloration. To conclude, our results provided useful genetic resources for future studies regarding the genetic mechanisms of skin coloration in leopard coral grouper, and also to assist breeders to conduct molecular assisted selection in leopard coral grouper farming.

## Methods

### Sample collection

Fish were wild-caught from the exclusive economic zone of Penghu Sea in Taiwan. Skin samples were immediately immersed in the RNA keeper reagent (Protech, Taiwan), and further stored at -80℃. Six fish with different skin colors, including three red individuals and three brown individuals, were selected for RNA extraction. The average body weight of fish was 0.7 ± 0.6 kg, and average body length was 38.1 ± 9.3 cm.

### RNA isolation

Total RNA was extracted from the fish skin tissue. The skin sample was homogenized in 1 mL of TRIzol reagent (Invitrogen, USA) containing stainless steel beads, following the manufacturer’s protocol. The quality and quantity of total RNA were determined by a DS-11 spectrophotometer (DeNovix, USA), and run on 1% agarose gel electrophoresis. The RNA samples were stored at -80℃ for further experiments.

### Library construction and RNA sequencing

RNA extracted from fish skin tissue was prepared for library construction. The RNA integrity was assessed by a Qsep400 (BiOptic, Taiwan) with quality number (RQN) values ≥ 7.0. A paired-end (PE) sequencing library was constructed using Illumina TruSeq RNA Library Prep Kit v2 (Illumina, USA), following the manufacturer’s protocol. The obtained library was sequenced using the Illumina NovaSeq 6000 platform (Illumina, USA) with 2 × 150 bp PE reads.

### Transcriptome assembly

To eliminate errors, adapters and low-quality reads from the raw data were removed by fastp [65]. The Phred score (Q20 and Q30) and GC content of the clean data were calculated. Sequence quality metrics were assessed using FastQC (http://www.bioinformatics.babraham.ac.uk/projects/fastqc/) software. Subsequently, all clean reads were aligned to the reference *P. leopardus* genome [31] using STAR [66]. The coordinate sorted bam file was assembled using Trinity program [67] for reference-guided *de novo* assembly. While the assembled data contains abundant duplicate transcripts, CD-HIT-EST clustering application [68] was used to remove redundant transcripts with 95% identity, and establish the final assembled transcripts. Potential protein-coding genes were identified from assembled transcripts using Transdecoder (https://github.com/TransDecoder/TransDecoder).

### Gene annotation

Transcriptome assembly annotation was performed with Trinotate pipeline (http://trinotate.github.io). The assemble transcripts were annotated using blastp and blastx program (http://www.ncbi.nlm.nih.gov/BLAST/) with E-value cutoff of 1e-10 against UniprotKB. Functional and pathway annotations were carried out via GO, KO (http://www.genome.jp/kegg) [69], and COG (http://www.ncbi.nlm.nih.gov/COG) databases.

### Differential expression analysis

Each PE read from the RNA-Seq was mapped with Bowtie2 [70] to the final assembled transcripts, and the quantification of the read count was generated using RSEM [71]. The read counts were normalized by the TMM method implemented in the R package “edgeR” [72], and generated PCA and heatmap. Differential expression analysis was performed using “edgeR”, and p-values were adjusted using the Benjamini-Hochberg (BH) method to control the FDR. Genes with FDR < 0.05 and an absolute value of log_2_ FC ≥ 2 were considered to be DEGs, and were used in the subsequent analysis. The GO and KEGG enrichment analysis of DEGs were performed using KOBAS [73].

### Quantitative real-time polymerase chain reaction (qRT-PCR)

cDNA synthesis was generated from total RNA using SuperScript® IV First-Strand cDNA Synthesis Reaction (Invitrogen, USA), and random hexamer primers (Invitrogen, USA). Diluted cDNA (10 ng/µL) was used as a template for qRT-PCR. β-actin was used as the housekeeping gene for the normalization of gene expression levels. The primers used for target and reference genes were designed based on transcriptome sequence using Primer-BLAST [74] with an amplicon size of approximately 70–240 bp. qRT-PCR analysis was performed in the QuantStudio™ 3 real-time PCR systems (Thermo Fisher Scientific, USA) and the reactions were carried out using PowerUp™ SYBR™ Green Master Mix (Thermo Fisher Scientific, USA). The conditions for reactions were 50℃ for 2 min, 95℃ for 2 min, followed by 40 cycles of 95℃ for 15 s, 60℃ for 15 min, 72℃ for 1 min. To ensure a single amplicon reaction, a melting curve analysis was performed at the end of each run. All the experiments were conducted with three biological replicates, and each group was conducted with four samples. The relative gene expression was calculated using the comparative threshold cycle (2^−△△CT^) method [75]. Student t-test was performed to determine the significant differences between red and brown groups. The Pearson correlation coefficient between RNA-Seq and qRT-PCR was calculated using “cor.test” function in R.

## Supplementary Information

**Additional file 1:** **Fig. S1.** COG classification analysis. **Fig. S2.** GO categorization of the annotation genes. **Fig. S3.** KO classification.** Fig. S4.** Differential expression analysis. **Fig. S5.** GO enrichment of DEGs between red and brown groups.** Fig. S6.** KEGG pathways enrichment analysis of DEGs between red and brown groups.

**Additional file 2:** **Table S1.** Heatmap analysis of top 230 DEGs (115 up regulation and 115 down regulation).

## Data Availability

The Sequence Read Archive (SRA) has been deposited at GenBank in NCBI. The datasets generated during the current study are available in the NCBI GenBank repository, with accession number PRJNA826254. Please see link below for details (https://www.ncbi.nlm.nih.gov/bioproject/PRJNA826254).
