# Correction: Comparative transcriptome analysis of skin color-associated genes in leopard coral grouper (*Plectropomus leopardus*)

**DOI:** 10.1186/s12864-023-09153-3

**Published:** 2023-02-10

**Authors:** Hung-Yi Wu, Kao-Sung Chen, You-Syu Huang, Hern-Yi Hsieh, Hsin Yuan Tsai

**Affiliations:** 1 Department of Marine Biotechnology and Resources, National Sun Yat-Sen University, Kaohsiung City, Taiwan; 2 Institute of Fisheries Science, National Taiwan University, Taipei City, Taiwan; 3 Planning and Information Division, Fisheries Research Institute, Council of Agriculture, Keelung, Taiwan; 4 Eastern Marine Biology Research Center, Taitung City, Taiwan; 5 Penghu Marine Biology Research Center, Penghu County, Magong, Taiwan; 6 Doctoral Degree Program in Marine Biotechnology, National Sun Yat-Sen University, Kaohsiung City, Taiwan

**Correction: BMC Genomics 24, 5 (2023)**

**https://doi.org/10.1186/s12864-022-09091-6**

Following the publication of the original article [1], the authors reported that one affiliation for the 2^nd^ author was omitted from the **Author Information** section. All affiliations have been updated.

The original article [1] has been corrected.
