# Supplementary material for: Comparative transcriptome analysis of skin color-associated genes in leopard coral grouper (Plectropomus leopardus)
Source: BMC Genomics. 2023 Jan 5;24:5. doi: 10.1186/s12864-022-09091-6 (PMC9817277; doi:10.1186/s12864-022-09091-6)
Supplement: Supplementary file 1 — Additional file 1: Fig. S1. COG classification analysis. Fig. S2. GO categorization of the annotation genes. Fig. S3. KO classification. Fig. S4. Differential expression analysis. Fig. S5. GO enrichment of DEGs between red and brown groups. Fig. S6. KEGG pathways enrichment analysis of DEGs between red and brown groups. [file 12864_2022_9091_MOESM1_ESM.pdf]

Supplementary Materials

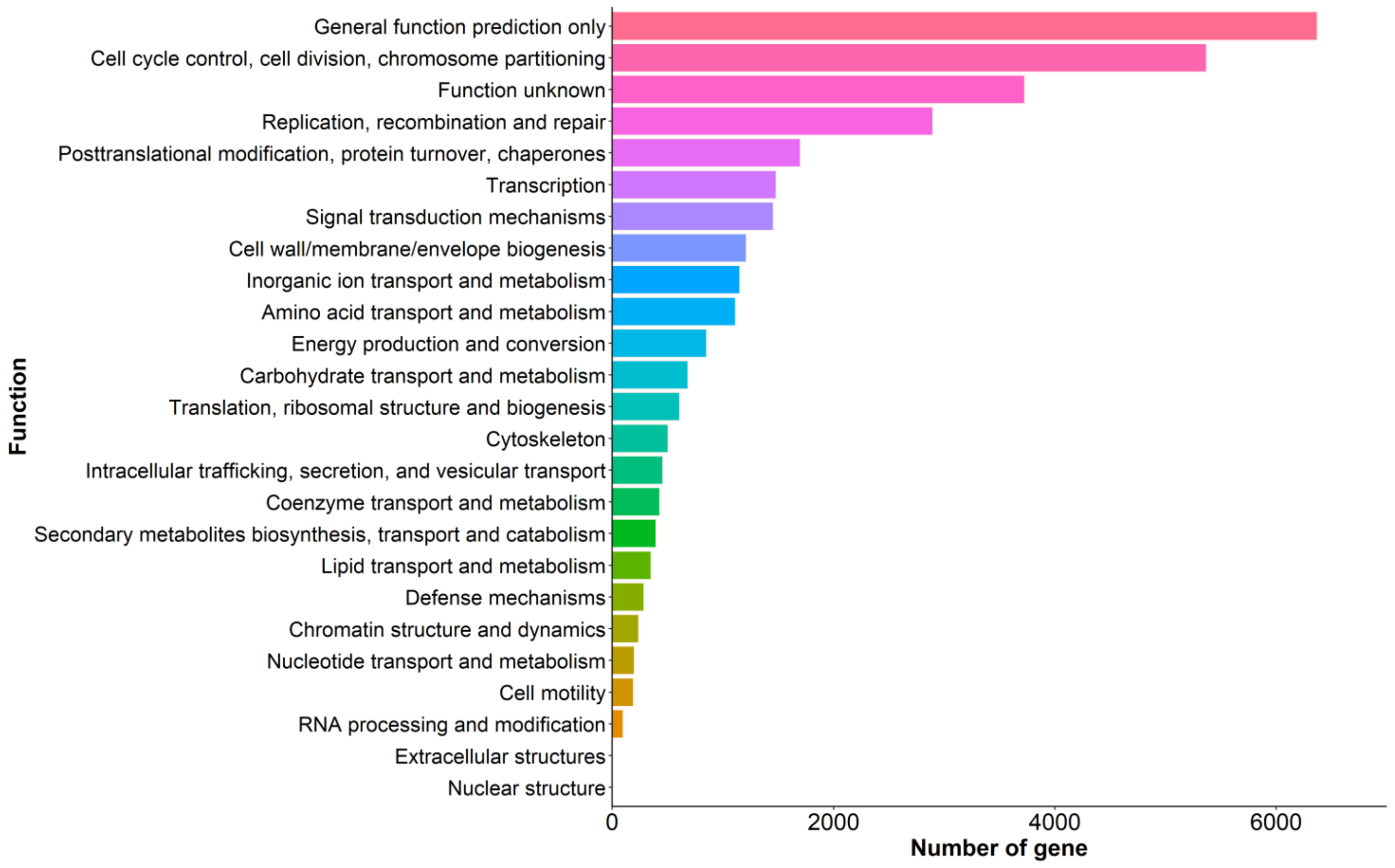

Fig. S1 COG classification analysis.

The Y-axis shows the function class, and the X-axis shows the number of gene in a definite functional cluster.

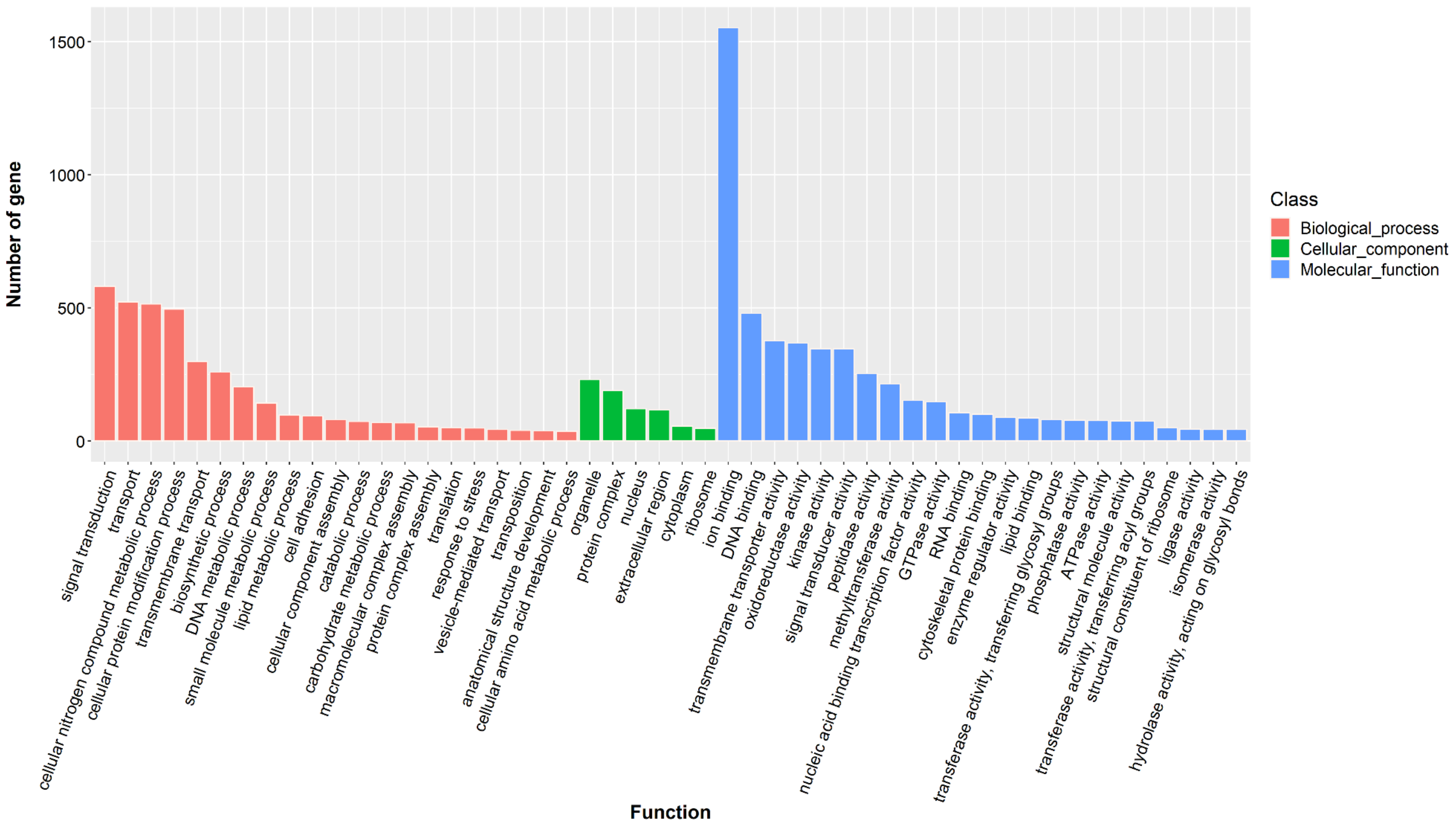

Fig. S2 GO categorization of the annotation genes.

The Y-axis shows the number of transcripts in a definite function, and the X-axis shows three major function categories of GO terms.

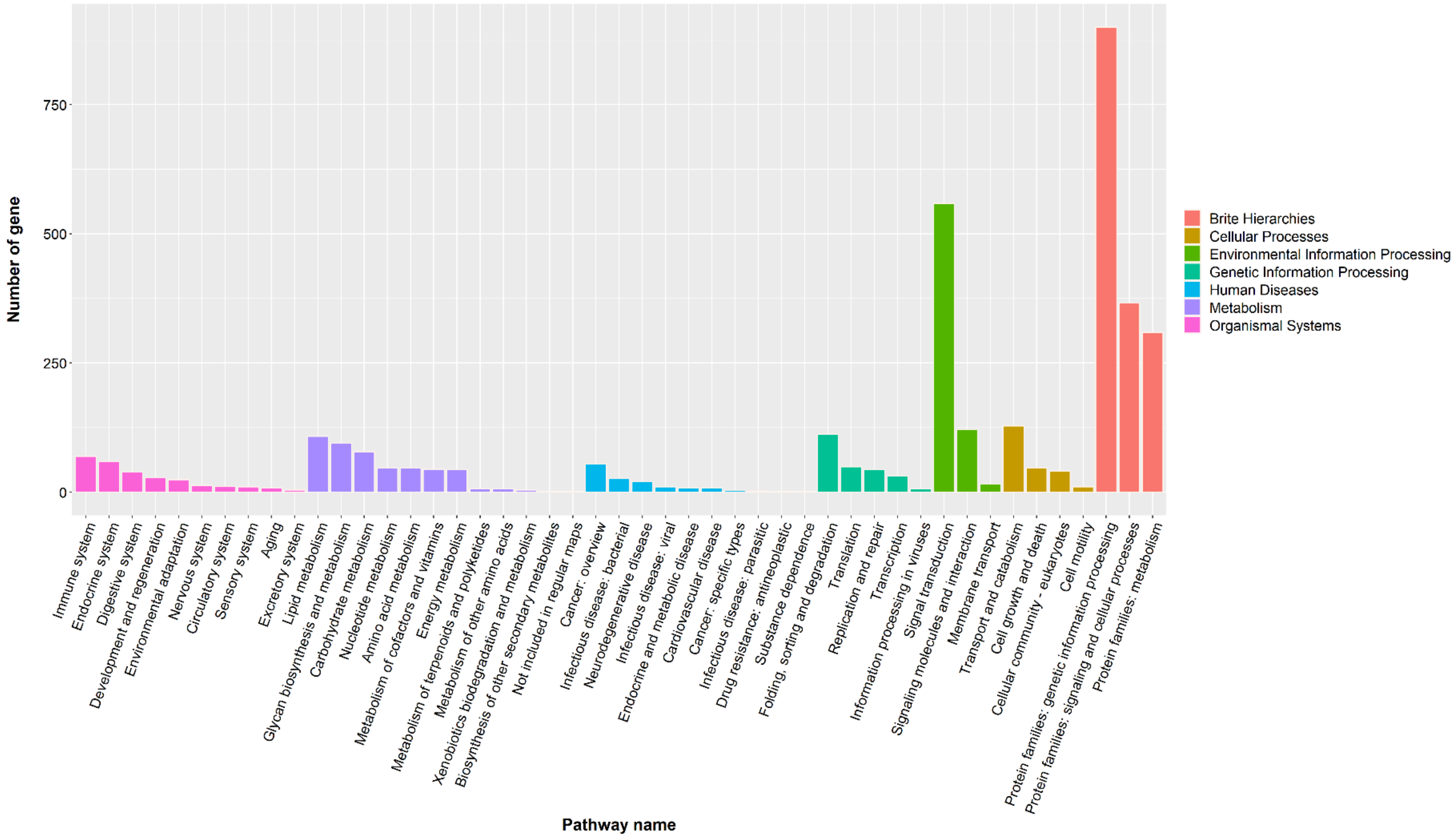

Fig. S3 KO classification.

KO analysis was classified into 7 categories including 47 pathways. The Y-axis shows the number of gene annotated in the pathway, and the X-axis shows the name of the KEGG pathway.

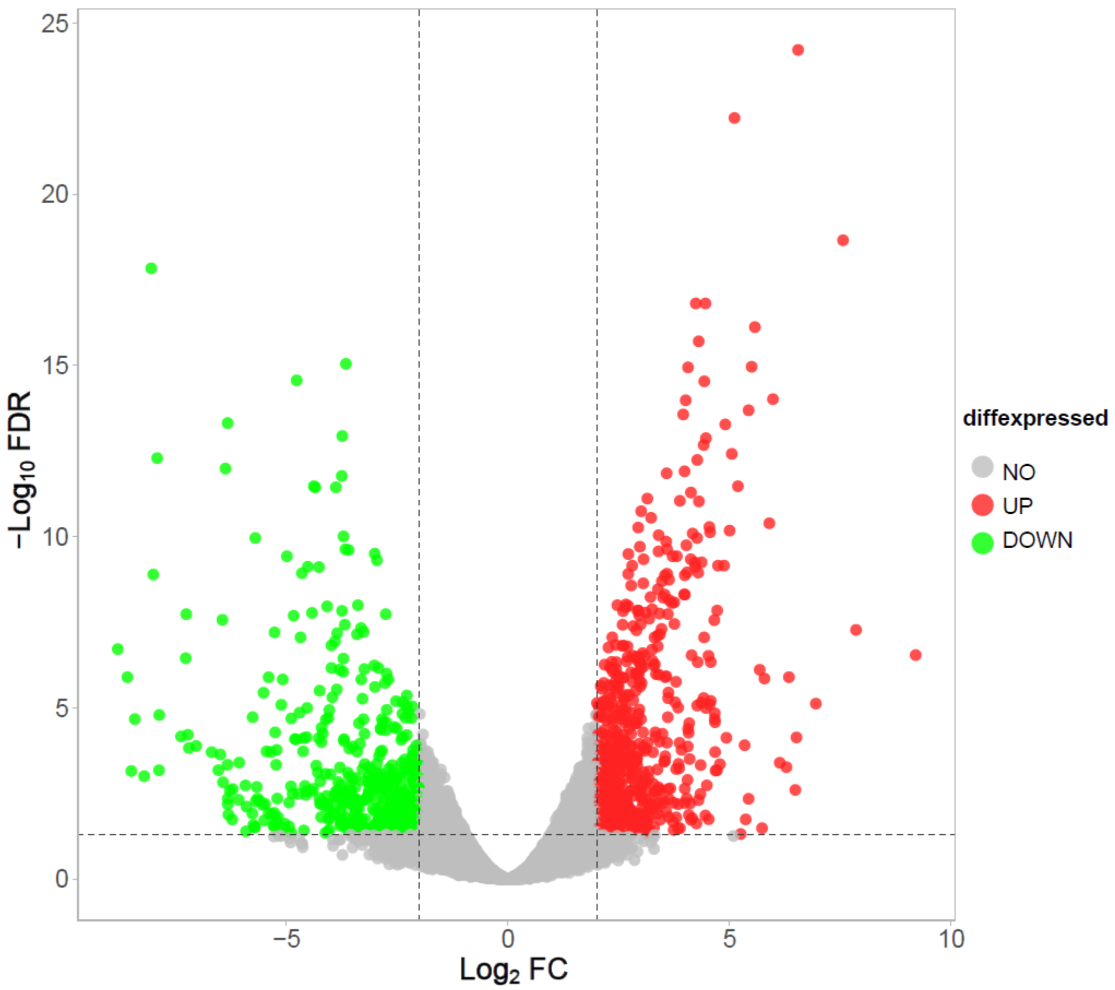

Fig. S4 Differential expression analysis.

Differential expression between red skin (R1, R2, and R3) versus brown skin (B1, B2, and B3). Red points represent up-regulated genes in red skin; green points represent up-regulated genes in brown skin. Grey points indicated genes that do not show significant differential expression.

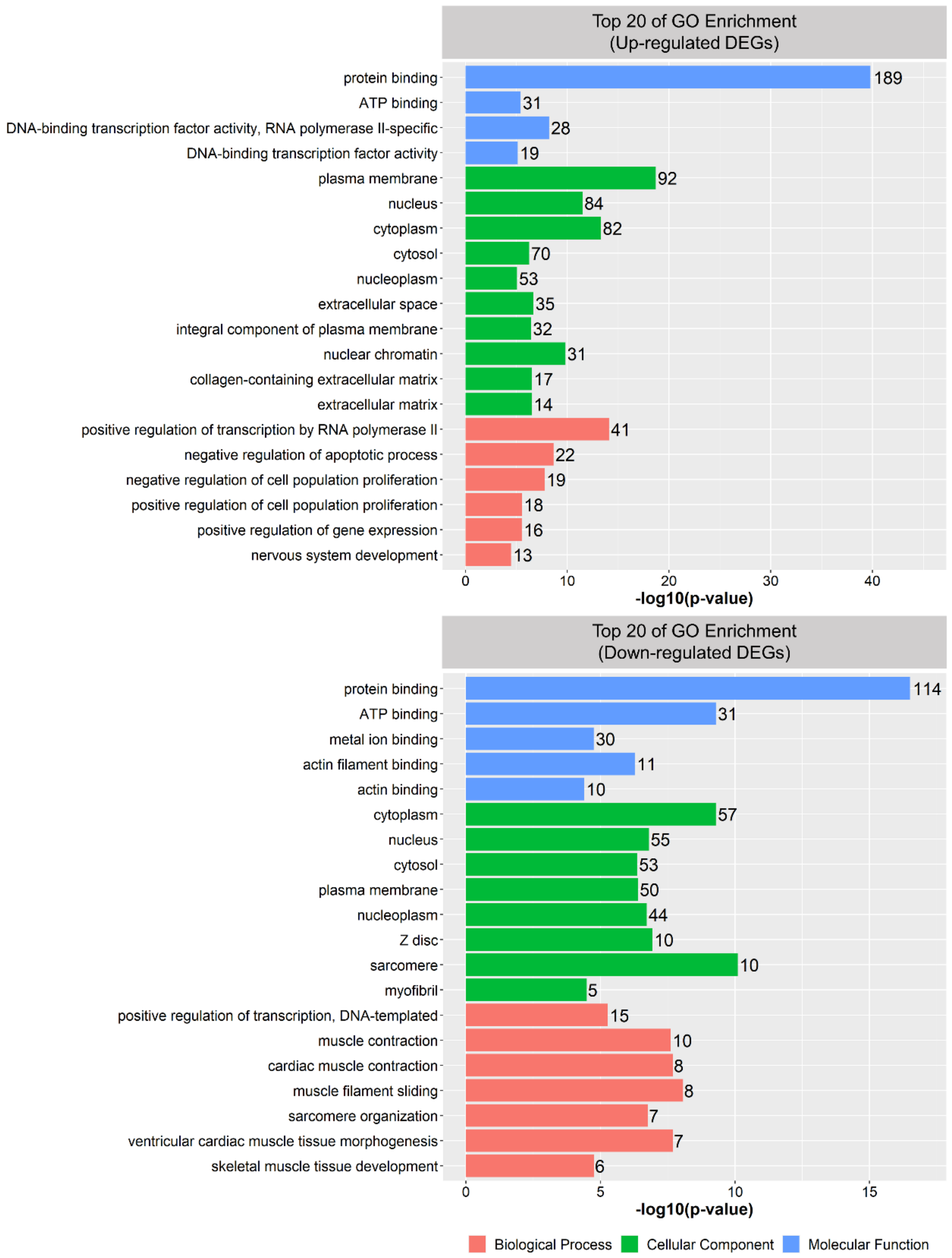

Fig. S5 GO enrichment of DEGs between red and brown groups.

The Y-axis represents three major function categories of GO terms, and the X-axis represents the enrichment score of the GO term. The top 20 GO enrichment for Up-regulated and down-regulated DEGs are shown. Different colors represent three main categories, namely, biological process (red), cellular component (green), and molecular function (blue).

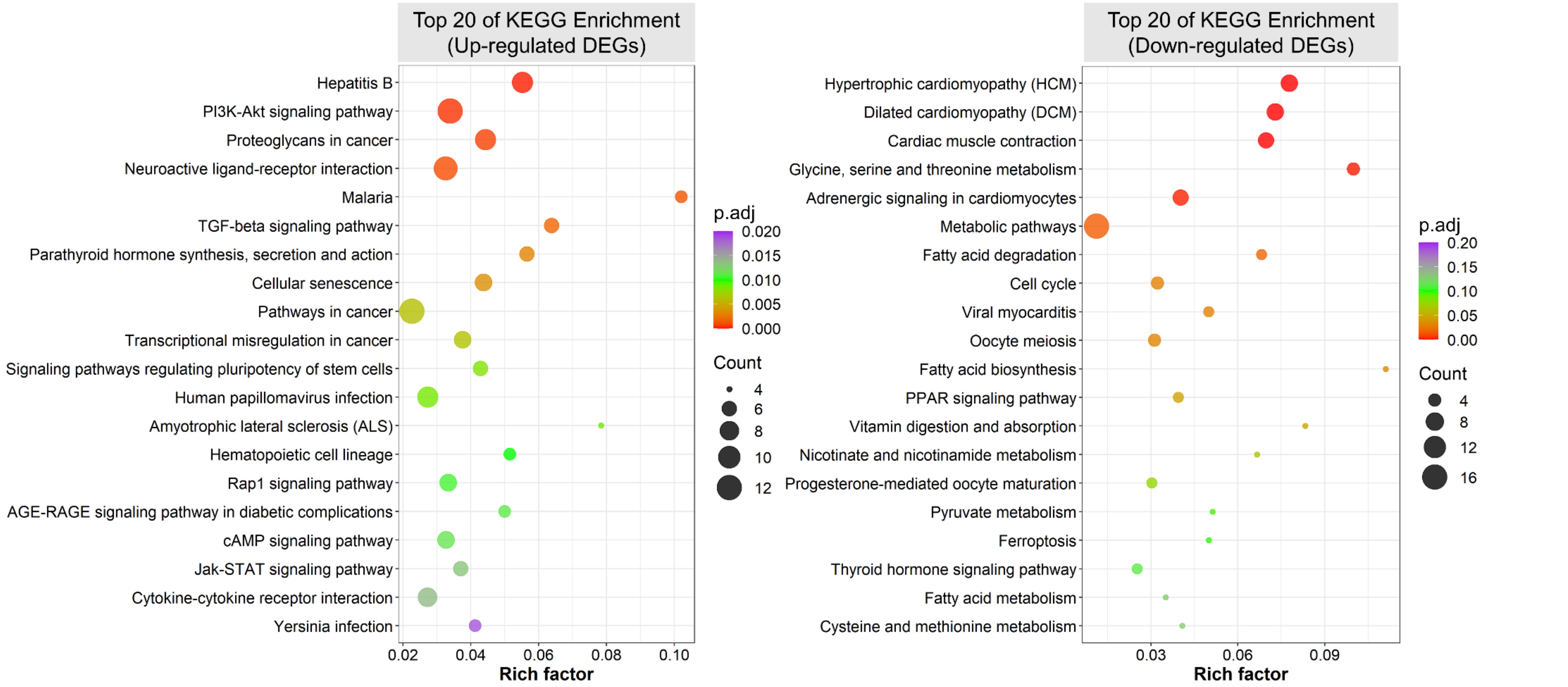

Fig. S6 KEGG pathways enrichment analysis of DEGs between red and brown groups.

The rich factor is the ratio of the number of enriched DEGs to total genes in the KEGG pathway. The color bar indicates the p-value corrected by Benjamini and Hochberg (BH) method, and the dot size indicates the number of DEGs. The top 20 KEGG pathways for up-regulated and down-regulated genes are shown.
